# Healthcare Utilization and Impact of Antifungal Stewardships Within Respiratory Care Settings: A Systematic Literature Review

**DOI:** 10.1007/s11046-021-00547-z

**Published:** 2021-05-15

**Authors:** Salma Aldossary, Anand Shah

**Affiliations:** 1 Respiratory Medicine, Royal Brompton and Harefield NHS Foundation Trust, London, UK; 2 MRC Centre of Global Infectious Disease Analysis, Department of Infectious Disease Epidemiology, School of Public Health, Imperial College London, London, UK

**Keywords:** Stewardship, Antifungal resistance, *Aspergillus fumigatus*, Antifungal, Chronic, Respiratory disease

## Abstract

**Introduction:**

Fungal infection and sensitization are common in chronic respiratory patient populations such as bronchiectasis, chronic obstructive pulmonary disease (COPD), and cystic fibrosis (CF) and are often associated with prolonged antifungal therapy (Hohmann et al. in Clin Infect Dis 15:939–940, 2010; Vissichelli et al. in Infect Prev Pract 1:100029, 2019), morbidity, and mortality. Although the use of antifungal stewardship (AFS) is increasing within an invasive fungal disease setting, its use and impact within a chronic respiratory setting have not been defined.

**Methods:**

A systematic literature review was conducted using PRISMA guidelines to evaluate the use of antifungal stewardship within a chronic respiratory care setting. Three databases have been searched, Medline via Ovid, Embase and GlobalHealth, for papers published between 1949 and 2020.

**Results:**

The initial search identified 987 papers from Medline, 1761 papers from Embase, and 481 papers from GlobalHealth. Only 28 papers met the criteria for inclusion in this systematic literature review. The included studies were subjected to CASP and GRADE assessments to rank their quality and applicability. Only two studies were focussed on *Aspergillus* species infection.

**Conclusion:**

Although antifungal stewardship is increasing, its applications are still limited in chronic respiratory care settings despite the prolonged requirement for antifungal therapy and high antimicrobial resistance.

**Supplementary Information:**

The online version contains supplementary material available at 10.1007/s11046-021-00547-z.

## Introduction

Individuals with chronic respiratory diseases are susceptible to fungal infection and allergy, which can cause a spectrum of diseases depending on the underlying host response. This can typically vary from sensitization, allergic bronchopulmonary aspergillosis (ABPA), chronic pulmonary aspergillosis (CPA), semi-invasive and invasive infection [1, 2]. There is a large global burden of fungal-related chronic lung disease, with global estimates of CPA at ~ 3 million cases [3, 4]. There is furthermore a recent appreciation of the impact of fungal infection and sensitization on morbidity of globally widespread chronic lung disease such as non-CF bronchiectasis and chronic obstructive pulmonary disease (COPD), which is additionally a significant risk factor for invasive fungal infection. *Aspergillus*-related disease also affects ~ 20% of individuals with cystic fibrosis (CF), leading to increased exacerbation frequency [3].

Oral triazole antifungal drugs are effective against *A. fumigatus* and are predominantly first-line therapy in the management of *Aspergillus*–related infection and allergy in chronic respiratory disease. However, there has been a rapid and global emergence of multiple triazole resistance phenotypes in *A. fumigatus* over the past decade, with a particularly high prevalence noted in patients with chronic lung disease [5]. Individuals with chronic lung disease and fungal infection/allergy often require a prolonged duration of antifungal therapy, with intra-pulmonary fungi within cavitatory or hypoxic microenvironments, likely predisposing to antifungal resistance development [6, 7]. Triazole resistance has been associated with increased mortality both within an invasive and chronic pulmonary aspergillosis setting [8].

Antifungal stewardship aims to provide a meaningful mechanism to guide medication prescription in these at-risk cohorts to improve outcome and reduce toxicity and emergence of antifungal resistance [9, 10]. The principle is based on existing antimicrobial stewardship programs (ASPs), which optimize antibiotic prescriptions by taking into account the range of action, pharmacokinetics, and pharmacodynamics (PK/PD) properties, length, and route of administration. Key to stewardship programs is therapy optimization with population level screening through regular therapeutic drug monitoring (TDM) to ensure that optimum PK/PD is achieved to increase effectiveness and reduce adverse outcomes and antifungal resistance [11, 12]. However, the impact and evidence-base for the use of antifungal stewardship in a chronic respiratory disease setting is not yet well defined. We perform a systematic literature review aiming to evaluate the current evidence of the use and impact of antifungal stewardship within a chronic respiratory disease setting.

## Methodology

A systematic literature review based on PRISMA guidelines was conducted [13]. Three databases have been searched, Medline via Ovid, Embase, and GlobalHealth, for papers published between 1949 and 2020. The search strategy (see Supplementary information) was designed to find papers that involve antifungal or antimicrobial stewardship.

- Inclusion criteria involve papers that implemented antifungal stewardship and reported the outcomes or studies that report clinical outcomes of antifungal therapeutic drug monitoring.
- Exclusion criteria involve papers (1) not in English, (2) case reports, (3) narrative or systematic reviews.

Studies were imported to Covidence for screening [14]. After screening, articles were removed for a number of reasons including no focus on antifungal agents or antifungal stewardship, wrong outcomes, wrong study design, systematic review or meta-analysis, not available, wrong patient population, wrong intervention or case reports. The PRISMA checklist was utilized in guiding the systematic literature review. Due to the differences in interventions, patient populations, and outcomes for the studies, the data were descriptively summarized. The findings were drawn after qualitative synthesis of data.

The quality of the included studies was determined using the GRADE (Grading of Recommendations, Assessment, Development, and Evaluation methodology), which is categorized to high, moderate, low, and very low [15, 16]. By definition, high classification indicates that further research is very unlikely to change the confidence in the estimate of effect. Moderate quality means further research is likely to have an important impact on the confidence in the estimate of effect. Low-grade classification indicates that further research is very likely to have an important impact, while very low classification indicates that an effective impact of the findings is very uncertain [15]. The studies collected for this review were tested using this approach to answer the clinical question on the usefulness and impact of the antifungal stewardship programs on patients in respiratory care.

**Fig. 1:**
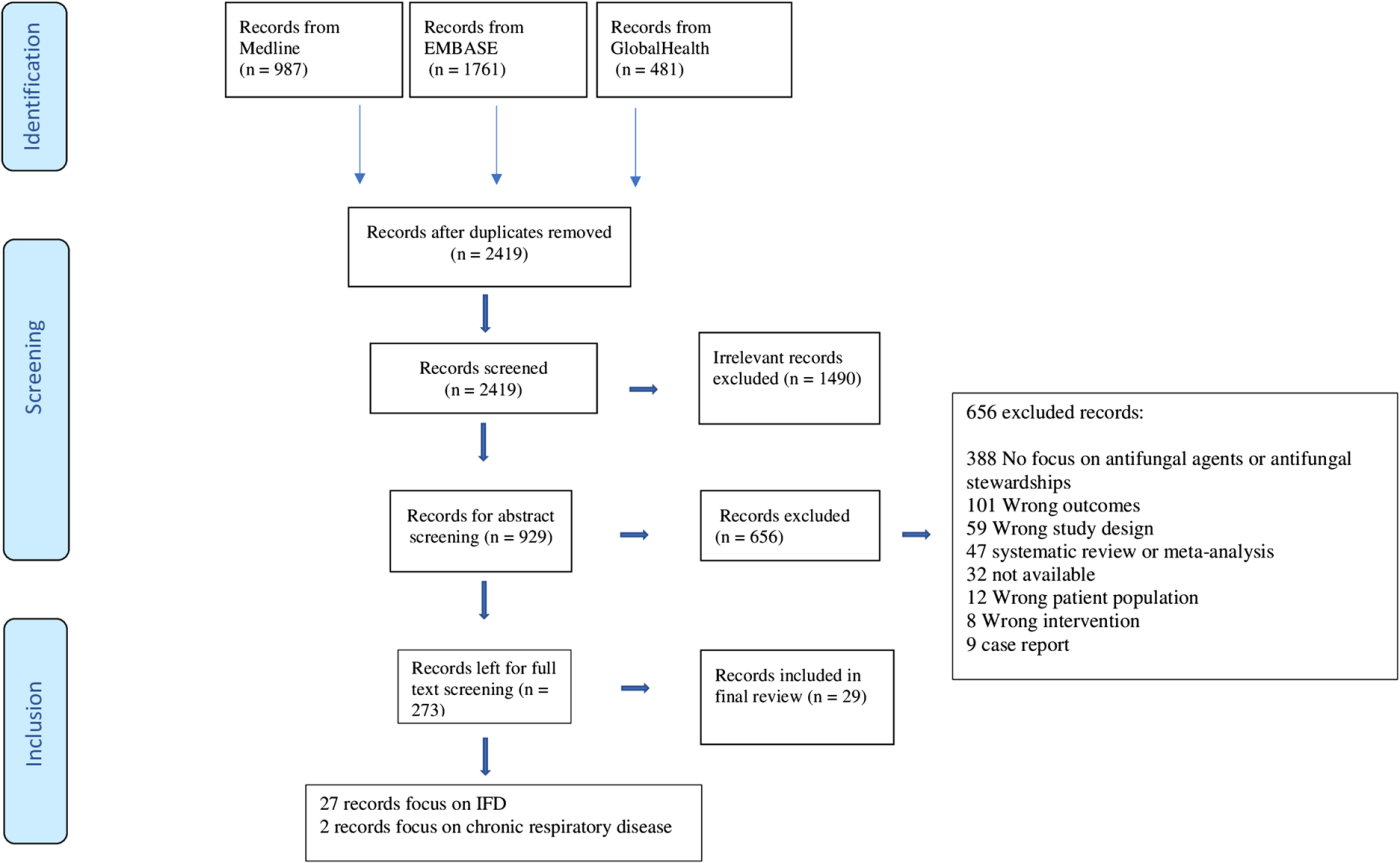
PRISMA flow diagram of search and selection process

**Table 1:** Included study characteristics

Study author, year	Study design	Type of fungal species	Intervention	Duration of intervention	Patient population, Settings	Presence of chronic respiratory patients	Outcomes after Stewardship	GRADE
Shah et al. [28]	Retrospective	*Candida* species	Invasive tests	48 h–24 days	Immunocompetent adult patients treated in the medical and surgical intensive care units	Yes	Fungal testing of respiratory tract specimens does not add diagnostic value	Low
Mondain et al. [29]	Prospective observational	Invasive aspergillosis and candidaemia	Multifaceted antifungal stewardship discussion	48 h–2 weeks	French teaching tertiary-care hospital	Yes	Antifungal stewardship programme was feasible, sustainable, and well accepted	Low
López-Medrano et al. [31]	Intervention	*Candida* species	Non-randomized uncontrolled before–after antimicrobial stewardship	1–2 years	The University Hospital 12 de Octubre. A 1300-bed hospital	Yes	Primary outcome of the study was a reduction in antifungal expenditure	Low
Alfandari et al. [20]	Retrospective	*Candida* species	Multidisciplinary collaborative use of antifungals in haematology	4–5 days	Allograft and acute leukemia induction chemotherapy patients. The Lille Regional Teaching Hospital	No	Decreased antifungal consumption, stabilized invasive fungal infections (IFI), and decreased IFI-related mortality	Low
Muñoz et al. [32]	Retrospective	*Candida* species	Educational and bedside intervention	2 years	Hospitalized patients with cases of invasive candidiasis and invasive aspergillosis	Yes	Reduced consumption of DDDs and reduced expenditure on antifungals	Low
Vissichelli et al. [2]	Retrospective	*Aspergillus* species	Review of bronchoalveolar lavage results	2 years	Autologous haematopoietic stem-cell transplant recipients	Yes	Antifungals were more likely to be escalated or changed	Low
Whitney et al. [24]	Retrospective	*Candida* species	Antifungal susceptibility testing	5–6 days	Adult in patients receiving amphotericin B, echinocandins, intravenous fluconazole, flucytosine, or voriconazole. St. George’s tertiary referral hospital	Yes	Antifungal expenditure initially reduced then increased to 20% above baseline over a 5-year period	Low
Hamada et al. [33]	Retrospective	N/A	Voriconazole trough concentration	4 days	Hospitalized patients on voriconazole, at five hospitals in Japan	No	Reduced incidence of hepatotoxicity	Low
Shah et al. [34]	Retrospective	*Candida* species	Administration of fluconazole or an echinocandin	5 + or − 2 days	Hospitalized patients with candidaemia	No	Less than 40% of echinocandin-treated patients with fluconazole-susceptible organisms were de-escalated to fluconazole	Low
Gurram et al. [43]	Retrospective	*Candida* species	Bronchoalveolar lavage	6–12 months	Adult immunocompromised patients who underwent bronchoscopy with Immunocompromised Host (ICH) Protocol	Yes	Patients with *Candida* sp. in bronchoscopy cultures are not likely to be intervened upon	Low
Rautemaa-Richardson et al. [35]	Retrospective	*Candida* species	Antifungal therapy	4 months	Patients prescribed micafungin for suspected or proven invasive candidosis. UK tertiary referral teaching hospital	Yes	Number of patients treated for invasive candidiasis decreased	Low
Mondain et al. [30]	Prospective observational	*Candida* species	Systematic evaluation	2–5 days	Teaching tertiary-care hospital	No	Improved quality of care and stable antifungal use and cost in the hospital	Very low
Antworth et al. [36]	Single-centre, quasi-experimental	*Candida* species	Comprehensive candidemia care bundle	3 days	Patients with candidemia. A 930-bed academic hospital	No	Improved management of patients with candidemia	Low
Menichetti et al. [21]	Retrospective	*Candida* species	Infectious disease consultation	30 days	Patients with documented candidemia cared for in Pisa tertiary care, University hospital	No	A lower 30-day in-hospital mortality rate for candidemia patients treated with infectious diseases consultation (IDC) with respect to those treated without	Moderate
Swoboda et al. [19]	Retrospective	*Candida* species	Standardized practice of antifungal therapy	45 days	Patients with an intensive care unit (ICU) stay of 1–24 h and either recovery of fungi from any site and/or application of systemic antifungals	No	Significant decrease in the use of antifungal agents as well as costs	Moderate
Browne et al. [9]	Retrospective	*Candida* species	Complex lung infection multidisciplinary meeting	6–12 months	Hospitalized patients with suspected fungal lung disease	Yes	Allowed a refinement in diagnosis of *Aspergillus*-associated lung diseases and improved stewardship of triazole drugs	Very Low
Reed et al. [37]	Quasi-experimental	*Candida* species	ASP pharmacist's intervention	1–2 years	The Ohio state Wexner Medical Centre, a 1229-bed teaching hospital	Yes	Timely notification from microbiology to the ASP PharmD in conjunction with ASP PharmD interventions resulted in more patients with candidemia receiving timely effective antifungal therapy	Moderate
Veringa et al. [38]	Retrospective	*Candida* species	Therapeutic drug monitoring (TDM) of anti-infective drugs	1–2 days	In-patients	No	TDM plays an important role in the optimization of treatment with anti-infective drugs	Low
Morris et al. [4]	Phased, multisite cohort	*Candida* species	Antimicrobial stewardship	3–5 days	Patients admitted to each ICU at the academic ICUs in Toronto, Canada	Yes	Sustained improvements in antimicrobial consumption and cost	Low
Pfaller and Castan Heira [39]	Prospective	*Candida* species	Rapid diagnostic testing and antifungal stewardship	24 h, 7 days	Hospitalized individuals	No	Improved care by increasing the awareness of candidiasis. Improve diagnostic efforts	Low
Märtson et al. [40]	Retrospective	*Candida* species	Therapeutic drug monitoring of posaconazole	1–3 years	Patients with haematological malignancies. The University Medical Center Groningen, the Netherlands	No	64% was the adequate posaconazole exposure; in the longitudinal analysis from all the confounders, only dose had a significant effect on posaconazole concentrations	Low
Kawaguchi et al. [41]	Single-centre, observational	*Candida* species	Antifungal stewardship programs (AFSPs)	1–3 years	Patients who received systemic antifungals at the Osaka City University Hospital (980-bed, tertiary-care teaching hospital)	Yes	As the appropriate selection of antifungals increases, a decrease in antifungal usage and cost reduction also occurs. This trend leads to improved prognoses of patients with candidemia	Low
Ito-Takeichi et al. [42]	Single-institutional prospective cohort	*Candida* species	1‐3, *β*-d-glucan (βDG) testing	1–2 days	In patients receiving intravenous antifungals at the 614-bed Gifu University Hospital	No	Parental antifungal use was significantly reduced	Low
Cavalieri et al. [46]	Retrospective	*Candida* species	MALDI-TOF mass spectrometry / Vitek 2	1–2 days	Hospitalized patients	Yes	An average 18 h faster microbial ID and antimicrobial susceptibility test results	Low
Steuber et al. [18]	Single-centre, retrospective, observational	*Candida* species	T2 Candida Panel (T2CP)	3 years	Patients with positive or negative T2CP at a 971-bed community hospital	No	Antifungal optimization occurred in 54% of patients who had antifungal orders at the time of T2CP test	Low
Hashemi et al. [45]	Retrospective	*Candida* species	Disc diffusion and micro-dilution	N/A	Clinical isolates of *Candida*	No	The *Candida* studied had the highest rates of sensitivity to caspofungin and amphotericin B. Among azoles, the highest sensitivity, respectively, is to miconazole, econazole, and then to fluconazole	Very low
Chabavizadeh et al. [44]	Retrospective	*Candida* species	Ganoderma lucidum (alcoholic extract)	N/A	Patients with candidemia admitted to some specialized hospitals in Tehran	No	*Ganoderma lucidum* can be used as an antifungal product in future studies for control and treatment of candidiasis	Very low
Nwankwo et al. [17]	Prospective	*Aspergillus*species	Antifungal stewardship team impact assessment	18 months	Chronic lung disease in patients and out-patients with fungal infections	Yes	The commonest infection was CPA, reduction in monthly antifungal expenditure as well as antifungal use after implementing AFS without increase in mortality/morbidity	Low

## Results

The primary search yielded 3229 papers (987 papers from Medline, 1761 papers from Embase, and 481 papers from GlobalHealth). As illustrated in Fig. 1, only 29 papers met inclusion criteria for this systematic literature review (see Supplementary information for references for all included papers). Table 1 provides a summary of the included papers.

- Disease area of focus

The predominant fungal infection studied in the eligible articles with inclusion of patients with underlying chronic respiratory disease was *Candida* species with 25 out of 29 articles focussing on invasive fungal disease infection (IFI). Although fourteen studies included sample populations consisting of patients with chronic respiratory illnesses, only two studies (Nwanko et al. and Browne et al.) had a focus specifically on chronic respiratory fungal infections and pulmonary aspergillosis, indicating a gap in existing knowledge/evidence base in the use of antifungal stewardship in this field [9, 17]. Although focussed in an invasive fungal infection setting, the implementation of antifungal stewardship was shown to be an effective intervention in a number of the included studies, with some (*n* = 3) having a moderate GRADE quality of evidence.

Of the two studies that analysed implementation of antifungal stewardship in a chronic respiratory disease setting, Nwanko et al. conducted a prospective cohort study over a 18-month period with 178 patients to determine the impact of an antifungal stewardship team in patients with chronic lung diseases at a tertiary cardio-pulmonary hospital [17]. The researchers implemented an antifungal stewardship team consisting of a mycologist and pharmacist provided weekly stewardship rounds, a multidisciplinary meeting twice a week and an outpatient clinic. The most common underlying fungal disease amongst the cohort was CPA by 32%. The study demonstrates through stewardship implementation the delivery of 285 specific recommendations to improve outcome. A statistical increase in therapeutic azole dosing was noted as a result of stewardship implementation alongside a significant 44% reduction in monthly antifungal expenditure, with no deleterious effect on outcome.

The study by Browne et al., in which complex fungal lung infections, defined as sub-acute invasive aspergillosis (SAIA), CPA, CPA/ABPA overlap, and ABPA/bacterial infection overlap, were managed through a novel MDT, which comprised three respiratory physicians, an infectious disease specialist, a microbiologist and two pharmacists. They met six times in a 12-month period to review patients on antifungal medication with chronic lung infection [9]. Out of the 32 cases the team discussed, 13 were categorized as complex fungal lung infection. Therapeutic drug monitoring was implemented for these cases and a significant reduction in development of resistance was noted.

- Quality of study analysis

The quality of the included studies was determined using the Grading of Recommendations, Assessments, Development, and Evaluations (GRADE) methodology, a widely used systemic approach to making clinical recommendations. The studies collected for this review were tested using this approach to answer the clinical question on the usefulness and impact of the antifungal stewardship programs on patients in respiratory care. According to the GRADE classification, the majority of the included records start with very low and low classification as the majority of them are observational studies. Three studies reached moderate GRADE quality classification, however, due to the impact of findings. Studies’ characteristics are shown in Table 1.

- Reported outcomes:

Optimization of antifungal use: Eleven articles concluded that antifungal stewardship is associated with increased appropriate use of medication and a reduction in the overuse/misuse of antifungal therapy. The reduction of unnecessary antifungal use importantly did not negatively affect the quality of care or patient outcomes.

Mortality/morbidity: within the papers identified by the systematic review, some of the intervention measures initiated in the systematic literature review such as optimizing diagnosis through novel rapid diagnostics (e.g. T2CP and use of MALDI-TOF mass spectrometry) resulted in quicker diagnosis and reduced inappropriate antifungal use [18]. Integrated multidisciplinary antifungal stewardship programmes were associated with a reduction in incidence in antifungal-attributed toxicities such as hepatotoxicity, as well as a reduction in 30-day in-hospital mortality rates for candidaemia [19–21].

## Discussion

In this systematic review to evaluate the evidence base for antifungal stewardship in a chronic respiratory disease setting, we show that the majority of the studies performed to date are focussed in an invasive fungal disease setting, predominantly with *Candida* species infection, with only four studies reaching moderate grade evidence. There is nevertheless evidence in this setting indicating the effectiveness of antifungal stewardship as an implementation strategy, with improved appropriate use of optimal antifungal therapy and evidence to suggest benefit in reducing attributable mortality. With clear protocols for indication and dosage alongside therapeutic drug monitoring of antifungal medication, there was improved optimization of antifungal therapy, with corresponding reductions in drug toxicity. It is unclear as yet, whether long-term, improved delivery of optimal antifungal therapy can reduce acquisition or development of antimicrobial resistance.

Our systematic literature review, however, showed very limited current evidence within a chronic respiratory care setting. Only two studies were focussed on chronic lung disease patients with fungal infection. Both of these studies confirm the susceptibility of chronic respiratory patients to fungal infection. However, there are significant limitations in the evidence on antifungal stewardship application in a chronic respiratory care setting [22, 23, 24]. Two of the included studies (Nwanko et al. and Browne et al.) are classified as low-quality evidence according to GRADE classification [9, 17]. The prospective and retrospective studies highlighting antifungal stewardship in respiratory care settings may contain bias given the small sample size utilized in both studies with imprecisions due to a confidence interval of less than 95% in the results obtained.

Nevertheless, according to several of the included studies, antifungal stewardship is an effective means of regulating antifungal use, advocating for optimal use of the drugs, and reducing unintended harm. Given the duration of antifungal therapy often required within a chronic respiratory setting, this indicates significant potential benefit from stewardship implementation. However, as yet, there is a lack of evidence, and given the high burden of disease with rising antimicrobial resistance levels, there is an urgent requirement for a robust systematic evidence base for widespread adoption. Although antifungal medication is used widely within chronic respiratory fungal allergic diseases with a number of studies highlighting the impact of fungal sensitization on chronic respiratory disease, none of the papers within our systematic review have analysed the effects of antifungal stewardship implementation on fungal sensitization control. Previous studies have suggested the importance of optimal azole therapeutic dosing within ABPA, and this is again a topic that requires further systematic prospective evidence base [25].

To date, there is little evidence on optimal antifungal management of fungal disease in a chronic respiratory setting, with significant variability in management and diagnosis. A survey on laboratory setting capability for fungal testing in the UK in 2017 found that the practice remained sub-optimal, with a lack of a standardized approach to fungal testing unlike bacterial infections and variable testing and documentation of antifungal resistance [26, 27]. We highlight in this systematic review the significant benefits following implementation of antifungal stewardship in an invasive fungal disease setting, with a lack of evidence base for implementation in a growing burden in a chronic respiratory disease setting with high antifungal resistance prevalence.

## Conclusion

This systematic literature review shows that antifungal stewardship is an essential extension of ASPs and presents many of the same benefits for patients in an invasive disease setting. However, the application and evidence base for antifungal stewardship are still limited in a chronic respiratory disease setting despite a high burden of fungal disease and antifungal resistance. Further research is urgently needed to understand the factors that lead to the development of antifungal resistance and assess the impact of antifungal stewardship within a chronic respiratory disease setting.

## Supplementary Information

Below is the link to the electronic supplementary material.

Supplementary file1 (DOCX 21 kb)

## Abbreviations

PRISMA: Preferred Reporting Items for Systematic Reviews and Meta-Analyses
ASP: Antimicrobial stewardship program
AFS: Antifungal stewardship
CASP: Critical Appraisal Skills Programme
GRADE: Grading of Recommendations Assessment, Development and Evaluation
ABPA: Allergic bronchopulmonary aspergillosis
TDM: Therapeutic drug monitoring
MDT: Multidisciplinary team
