# Supplementary material for: Healthcare Utilization and Impact of Antifungal Stewardships Within Respiratory Care Settings: A Systematic Literature Review
Source: Mycopathologia. 2021 May 15;186(5):673–84. doi: 10.1007/s11046-021-00547-z (PMC8536614; doi:10.1007/s11046-021-00547-z)
Supplement: Supplementary file 1 — Supplementary file1 (DOCX 21 kb) [file 11046_2021_547_MOESM1_ESM.docx]

# Supplementary information: Healthcare utilisation and impact of antifungal stewardships within respiratory care settings: a systematic literature review

- Salma Aldossary^1^, Anand Shah^1,2^

Affiliations:

1. Respiratory Medicine, Royal Brompton and Harefield NHS Foundation Trust, London, UK
2. MRC Centre of Global Infectious Disease Analysis, Department of Infectious Disease Epidemiology, School of Public Health, Imperial College London, UK.

Corresponding author: Dr Anand Shah, Consultant Respiratory Physician, Royal Brompton and Harefield NHS Foundation Trust, London, UK. Email: [s.anand@imperial.ac.uk](mailto:s.anand@imperial.ac.uk); ORCID: 0000-0001-5257-520X

**Supplementary methods:**

Search Strategy:

1 ((antifungal or antimicrobial or fungal) adj3 (stewardship or guideline* or programme*)).mp. [mp=abstract, title, original title, broad terms, heading words, identifiers, cabicodes] (2639)

1. 2  limit 1 to english language (2575)
2. 3  (drug therap* or treatment outcome* or medication review or therap* drug

monitor* or invasive fungal or fungal).mp. [mp=abstract, title, original title, broad terms, heading words, identifiers, cabicodes] (296570)

1. 4  limit 3 to english language (255761)
2. 5  (evidence based or antifungal management or evaluat* or drug utilization or

prognosis* or treatment outcome* or mycology or therapeutic drug monitor*).mp. [mp=abstract, title, original title, broad terms, heading words, identifiers, cabicodes] (666460)

1. 6  limit 5 to english language (578944)
2. 7  2 and 4 (1371)
3. 8  6 and 7 (481)

**References for papers included.**

1. Shah, D. N. *et al.* Evaluation of antifungal therapy in patients with candidaemia based on susceptibility testing results: Implications for antimicrobial stewardship programmes. *J. Antimicrob. Chemother.* (2011) doi:10.1093/jac/dkr244.

2. Mondain, V. *et al.* Impact of an antifungal stewardship programme in a teaching hospital: A prospective study. *Clin. Microbiol. Infect.* (2012).

3. Mondain, V. *et al.* A 6-year antifungal stewardship programme in a teaching hospital. *Infection* (2013) doi:10.1007/s15010-013-0431-1.

4. López-Medrano, F. *et al.* A non-compulsory stewardship programme for the management of antifungals in a university-affiliated hospital. *Clin. Microbiol. Infect.* (2013) doi:10.1111/j.1469-0691.2012.03891.x.

5. Alfandari, S., Berthon, C. & Coiteux, V. Antifungal stewardship: Implementation in a French teaching hospital. *Med. Mal. Infect.* (2014) doi:10.1016/j.medmal.2014.01.012.

6. Muñoz, P., Valerio, M., Vena, A. & Bouza, E. Antifungal stewardship in daily practice and health economic implications. *Mycoses* (2015) doi:10.1111/myc.12329.

7. Vissichelli, N. C. *et al.* Bronchoalveolar lavage to evaluate new pulmonary infiltrates in allogeneic hematopoietic stem cell transplant recipients: impact on antimicrobial optimization. *Infect. Prev. Pract.* (2019) doi:10.1016/j.infpip.2019.100029.

8. Whitney, L. *et al.* Effectiveness of an antifungal stewardship programme at a London teaching hospital 2010-16. *J. Antimicrob. Chemother.* (2019) doi:10.1093/jac/dky389.

9. Hamada, Y. *et al.* Effects of antifungal stewardship using therapeutic drug monitoring in voriconazole therapy on the prevention and control of hepatotoxicity and visual symptoms: A multicentre study conducted in Japan. *Mycoses* (2020) doi:10.1111/myc.13129.

10. Shah, A. S. *et al.* Fungal Diagnostic Stewardship in Bronchoscopy Specimens for Immunocompetent Patients in the Intensive Care Unit. *Mayo Clin. Proc.* (2019) doi:10.1016/j.mayocp.2019.02.020.

11. Rautemaa-Richardson, R. *et al.* Impact of a diagnostics-driven antifungal stewardship programme in a UK tertiary referral teaching hospital. *J. Antimicrob. Chemother.* (2018) doi:10.1093/jac/dky360.

12. M.M., C. *et al.* A comprehensive diagnostic approach using galactomannan, targeted beta-d-glucan, baseline computerized tomography and biopsy yields a significant burden of invasive fungal disease in at risk haematology patients. *Br. J. Haematol.* (2015).

13. Antworth, A. *et al.* Impact of an antimicrobial stewardship program comprehensive care bundle on management of candidemia. *Pharmacotherapy* (2013) doi:10.1002/phar.1186.

14. Menichetti, F. *et al.* Impact of infectious diseases consultation as a part of an antifungal stewardship programme on candidemia outcome in an Italian tertiary-care, University hospital. *J. Chemother.* (2018) doi:10.1080/1120009X.2018.1507086.

15. Swoboda, S. *et al.* Implementation of practice guidelines for antifungal therapy in a surgical intensive care unit and its impact on use and costs. *Chemotherapy* (2009) doi:10.1159/000264672.

16. Reed, E. E. *et al.* Improving the management of candidemia through antimicrobial stewardship interventions. *Diagn. Microbiol. Infect. Dis.* (2014) doi:10.1016/j.diagmicrobio.2013.11.012.

17. Veringa, A. *et al.* LC-MS/MS for Therapeutic Drug Monitoring of anti-infective drugs. *TrAC - Trends in Analytical Chemistry* (2016) doi:10.1016/j.trac.2015.11.026.

18. Morris, A. M. *et al.* Long-Term Effects of Phased Implementation of Antimicrobial Stewardship in Academic ICUs: 2007-2015∗. *Crit. Care Med.* (2019) doi:10.1097/CCM.0000000000003514.

19. Pfaller, M. A. & Castanheira, M. Nosocomial candidiasis: Antifungal stewardship and the importance of rapid diagnosis. *Medical Mycology* (2016) doi:10.1093/mmy/myv076.

20. Märtson, A. G. *et al.* Posaconazole therapeutic drug monitoring in clinical practice and longitudinal analysis of the effect of routine laboratory measurements on posaconazole concentrations. *Mycoses* (2019) doi:10.1111/myc.12948.

21. Kawaguchi, H. *et al.* The effects of antifungal stewardship programs at a tertiary-care teaching hospital in Japan. *J. Infect. Chemother.* (2019) doi:10.1016/j.jiac.2019.01.015.

22. Ito-Takeichi, S. *et al.* The impact of implementing an antifungal stewardship with monitoring of 1-3, β-D-glucan values on antifungal consumption and clinical outcomes. *J. Clin. Pharm. Ther.* (2019) doi:10.1111/jcpt.12809.

23. Steuber, T. D. *et al.* Utilization and impact of a rapid Candida panel on antifungal stewardship program within a large community hospital. *Diagn. Microbiol. Infect. Dis.* (2020) doi:10.1016/j.diagmicrobio.2020.115086.

24. Nwankwo, L. *et al.* A prospective real-world study of the impact of an antifungal stewardship program in a tertiary respiratory-medicine setting. *Antimicrobial Agents and Chemotherapy* (2018) doi:10.1128/AAC.00402-18.

25. Browne, A. *et al.* P138 Improving anti-fungal stewardship and the management of chronic pulmonary aspergillosis through a complex lung infection MDT. in (2019). doi:10.1136/thorax-2019-btsabstracts2019.281.

26. Gurram, P., Vashistha, K., O’Horo, J. C. & Shah, A. 267. Fungal Culture Diagnostic Stewardship: An Avenue for Antimicrobial Stewardship in the Immunocompromised Host. *Open Forum Infect. Dis.* (2019) doi:10.1093/ofid/ofz360.342.

27. Chabavizadeh J. The antifungal effects of alcoholic extract of Ganoderma lucidum on candida isolates. 2^nd^ International Conference on Mycology & Mushrooms (2017)

28. Hashemi J. Identification of species and evaluation drug susceptibility of Candida isolated from patients with visceral candidiasis by disk diffusion and micro-dilution methods. 5^th^ International Conference on Parasitology & Microbiology (2018)

29. Cavalieri et al. Matrix-Assisted Laser Desorption/Ionization-Time of Flight (MALDI-TOF) and Vitek 2 Along With Antimicrobial Stewardship (ASP) Result in Faster Antimicrobial Therapy for Infected Patients: The CHI Health Experience. [Open Forum Infect Dis](https://www.ncbi.nlm.nih.gov/pmc/articles/PMC6253327/). 2018 Nov; 5(Suppl 1): S513.
